# Supplementary material for: Atherogenic dyslipidemia and associated risk factors among hypertensive patients of five health facilities in Northeast Ethiopia
Source: PLoS One. 2023 Feb 14;18(2):e0277185. doi: 10.1371/journal.pone.0277185 (PMC9928121; doi:10.1371/journal.pone.0277185)
Supplement: Attachment — Submitted filename: Response to reviewers.docx [file pone.0277185.s002.docx]

Dear editors and reviewers, I greatly acknowledge your comments which are actually very crucial for the quality of the paper as well as for my future improvements.  As per your comments, all things are corrected and the corrected parts were alighted with green.

Major comments

**Reviewer point #1):** The primary objective and most important outcome of this study is the percentage of hypertensive people with dyslipidemia. The sample size was calculated to estimate this with a specific precision. Yet, no confidence intervals are presented around the point estimates of the actual percentage of hypertensive people with dyslipidemia. Such CIs are useful as they indeed show how precise the estimates for prevalence of the different dyslipidemia markers are.

**Author response:** Yes, we agreed with the reviewers, a 95% CI was calculated and indicated in the result part. Here is the calculated CI (93.2%; 95%CI: 90.6-95.6)

**Reviewer point #2):** Wording referring to the population (results, discussion): Sometimes “participants” is used, other times “people” or identifiers like “women”. None of these are technically correct. The population here is “people with hypertension in Northeast Ethiopia”, which is different from the sample (a subset of such people) or from “people”ingeneral. While it doesn’t need to be repeated constantly, some attention should be given throughout the manuscript to the fact that this analysis is representative for the proper population: people with hypertension in Northeast Ethiopia. This is correctly done in the abstract and conclusion.

**Author response:** Of course, this study was inferred to the hypertensive individuals so, as per the reviewer's comment we tried to replace some identifiers like “women, people, and participants with more appropriate descriptions like hypertensive individuals or people with hypertension.

**Reviewer point #3):** Material & Methods: sometimes too much details are given and information tends to get needlessly repeated Examples: definition of BMI (repeated twice, and common knowledge so not needed), multiple repeats on how height measurements are taken, paragraph on quality control can probably be shortened,

**Author response:** Here we accepted the reviewer's comment and it was due to clerical errors. Now we avoid repetitive descriptions and unnecessary details and it was shown in the manuscript.

**Reviewer point #4):** Table 5: please format the table better, it is hard to read like this. Table 5 and Table 6: please doublecheck all numbers carefully. Some results, while possible, are a bit strange. It is unclear to me how confidence intervals for logistic regression are calculated in SPSS, these results do not match what other statistical software could give. A few examples: 2.02(1.62-3.70) (maybe the lower bound is 1.26?); 1.33(1.08-1.40), 2.86(1.65-3.14) (does not make sense that the estimate is closer to the upper bound in these cases).

**Author response:** Based on the reviewer’s suggestion we tried to check all the numbers carefully. Since it was an error while copying numbers and now it was solved and corrected.

Reviewer point #5): Conclusion: “were significant determinants of dyslipidemia” Alas, you did it perfect during the whole paper, even correctly explicitly mentioning that a cross-sectional study cannot address causal links. Since no causality can be concluded, you should not state that these are “determinants” of dyslipidemia. Instead using “predictors” or “significantly associated” are both correct options.

**Author response:** Yes we accept the reviewer's comment and we corrected it following the reviewer's suggestion.

**Reviewer point #6):** I did not find a data sharing statement in the manuscript. Instead, when talking about data sharing, you replied "No - some restrictions will apply" and data available "On request". I understand that this is not a sufficiently detailed answer in light of the PLOS Data Policy. Please provide clear instructions on how a sufficiently anonymised version of the data can be obtained.
**Author response:** It was an error while submitting the manuscript online and I can assure you that all relevant data are included in the paper.

Minor comments:

**Reviewer point #7):** Abstract and page 7 (Data analysis): “Statistical significance was declared at p ≤0.05.” Please change to p < 0.05 (shouldn’t make a difference in results or interpretation).
**Author response:** Yes we have corrected p ≤0.05 was changed to p < 0.05
**Reviewer point #8):** Page 5: (Study Population and legibility Criteria) “All volunteer hypertensive patients (…) were eligible. However (…)” Please rewrite. The part following “however” makes it clear that not all patients were eligible.
**Author response:** Some physiological and pathological factors that could alter serum lipid profiles, such as pregnancy, taking lipid-altering drugs, and antihyperlipidemic medications, were excluded because the study was designed to assess dyslipidemia.

**Reviewer point #9):** Page 5: (Data collection and quality control) “Well-trained two data collectors” Rewrite to “Two well-trained data collectors”
**Author response:** Yes we have to rewrite it as per the reviewer's comment.
**Reviewer point #10):** Table 1: HDL-c <40 mg/dL vs. occupation: p-value is missing for this specific comparison
**Author response:** The missing p-value was putt as 0.21 in the space provided.
**Reviewer point #11):** Page 11, bottom: “the habit of not eating fruits and vegetables were substantially linked with elevated TC levels but not with elevated TG levels (Table 5).”
Replace substantially by significantly. Substantial would be linked to effect size, while this statement should only refer only to statistical significance.
**Author response:** Here we have replaced the word ‘substantially’ with significantly and you can see it in the manuscript.
**Reviewer point #12):** Page 14: “(AOR: 1.49, 95%CI: 127-1.91)”: add decimal sign in 1.27 “and 2.0 (AOR: 1.90, 95%CI: 1.51-2.52)”: change 2.0 by 1.9

**Author response:** Yes it was corrected.
