# Supplementary material for: Atherogenic dyslipidemia and associated risk factors among hypertensive patients of five health facilities in Northeast Ethiopia
Source: PLoS One. 2023 Feb 14;18(2):e0277185. doi: 10.1371/journal.pone.0277185 (PMC9928121; doi:10.1371/journal.pone.0277185)
Supplement: Attachment — Submitted filename: Response to editors and reviewers.docx [file pone.0277185.s003.docx]

Dear Editors and Reviewers,

As per your comments, everything was revised and fixed in the last submission. However, aside from the Data Availability Statement concern expressed by Reviewer 2, I couldn't find any particular concerns that still needed to be fixed and submitted with tracked modifications. I stated that there are some restrictions in my initial proposal. Naturally, it was a mistake that was done by the correspondence author alone, who neglected to pay attention during the online submission process. However, I had highlighted it in my prior responses to reviewers when I stated, "All the essential data are within the manuscript." Therefore, I have also includes the Data Availability Statement by saying ‘**All the relevant data are within the manuscript**’ in the manuscript.

Thank you very much for your consideration.

Your Sincerely,
