# Supplementary material for: Atherogenic dyslipidemia and associated risk factors among hypertensive patients of five health facilities in Northeast Ethiopia
Source: PLoS One. 2023 Feb 14;18(2):e0277185. doi: 10.1371/journal.pone.0277185 (PMC9928121; doi:10.1371/journal.pone.0277185)
Supplement: Attachment — Submitted filename: Response to editors and reviewers.docx [file pone.0277185.s004.docx]

Dear Editors and Reviewers,

**Reviewer point:** Have the authors made all data underlying the findings in their manuscript fully available?

**Author response:** Pardon me for the inconvenience. As per your comments, everything was revised and fixed in the last submission. However, I stated that there are some restrictions in my initial submission; naturally, it was a mistake that was done by the correspondence author alone, who neglected to pay attention during the online submission process. Now I assure you again there is no restriction regarding data availability at all. Therefore, I have also included the Data Availability Statement by saying; **we will provide repository information for our data at acceptance as Supporting Information files.** And I highlighted it with yellowish.

**Editor point:** Journal Requirements:

Please review your reference list to ensure that it is complete and correct. If you have cited papers that have been retracted, please include the rationale for doing so in the manuscript text, or remove these references and replace them with relevant current references. Any changes to the reference list should be mentioned in the rebuttal letter that accompanies your revised manuscript. If you need to cite a retracted article, indicate the article’s retracted status in the References list and also include a citation and full reference for the retraction notice.

Author response: I have already checked all the references, and it is complete and correct.

Thank you very much for your consideration.

Kind regards,

Ousman
